# Supplementary material for: All-optical diamond heater-thermometer enables versatile and reliable thermal modulation of ion channels at the single-cell level
Source: Biophys J. 2025 Nov 12;125(1):125–33. doi: 10.1016/j.bpj.2025.11.014 (PMC12821019; doi:10.1016/j.bpj.2025.11.014)
Supplement: Document S1. Figures S1 and S2 [file mmc1.pdf]

Biophysical Journal, Volume *125*

Supplemental information

All-optical diamond heater-thermometer enables versatile and reliable thermal modulation of ion channels at the single-cell level

Jean-Sébastien Rougier, Eugene Glushkov, Sabrina Guichard, Jan Kucera, Vadim Zeeb, and Hugues Abriel

All-optical Diamond Heater-Thermometer enables versatile and reliable thermal modulation of ion channels at the single-cell level

Jean-Sébastien Rougier[1*], Eugene Glushkov[2,3], Sabrina Guichard[1], Jan Kucera[4], Vadim Zeeb[2*], Hugues Abriel[1]

[1] Institute of Biochemistry and Molecular Medicine, University of Bern, Bern, Switzerland

[2] NanThermix SA, EPFL Innovation Park, 1015 Lausanne, Switzerland

[3] Laboratory of Nanoscale Biology (LBEN), EPFL, 1015 Lausanne, Switzerland

[4] Department of Physiology, University of Bern, Bern, Switzerland

*Corresponding email: jean-sebastien.rougier@unibe.ch, vadim@nanthermix.com

Supplementary

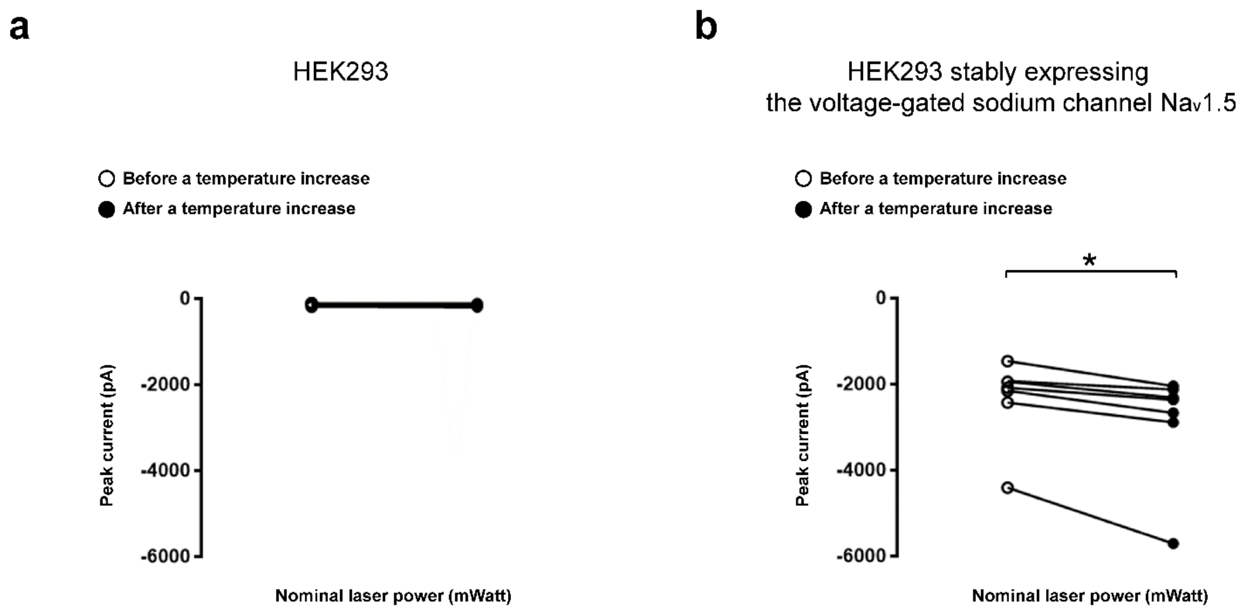

Figure S1. Only the voltage-gated sodium channels Nav1.5 are modulated by the local heat pulse: a) wild-type cells; b) cells stably expressing the voltage-gated sodium channels Nav1.5.

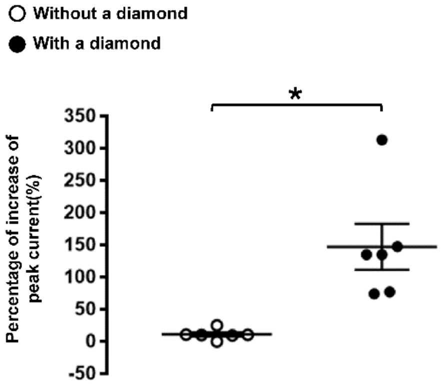

Figure S2. Laser illumination does not affect the biophysical properties of the Nav1.5 channel, as shown by the difference in the peak current increase when a pipette without (white circle) and with (black circle) a diamond is used at a laser power of 40 mW (*; $p < 0.05$).
