# Supplementary material for: All-optical diamond heater-thermometer enables versatile and reliable thermal modulation of ion channels at the single-cell level
Source: Biophys J. 2025 Nov 12;125(1):125–33. doi: 10.1016/j.bpj.2025.11.014 (PMC12821019; doi:10.1016/j.bpj.2025.11.014)
Supplement: Document S2. Article plus supporting material [file mmc2.pdf]

Biophysical Journal
Article

Biophysical Society

All-optical diamond heater-thermometer enables versatile and reliable thermal modulation of ion channels at the single-cell level

Jean-Sébastien Rougier,[1,*] Eugene Glushkov,[2,3] Sabrina Guichard,[1] Jan Kucera,[4] Vadim Zeeb,[2,*] and Hugues Abriel[1]
[1]Institute of Biochemistry and Molecular Medicine, University of Bern, Bern, Switzerland; [2]NanThermix SA, EPFL Innovation Park, 1015 Lausanne, Switzerland; [3]Laboratory of Nanoscale Biology (LBEN), EPFL, 1015 Lausanne, Switzerland; and [4]Department of Physiology, University of Bern, Bern, Switzerland

ABSTRACT A living cell is a nonequilibrium thermodynamic system where, nevertheless, a notion of local equilibrium exists. This notion applies to all micro- and nanoscale aqueous volumes, each containing a large number of molecules. This allows one to define sets of local conditions, including thermodynamic ones; for instance, a defined temperature requires thermodynamic equilibrium by definition. Once such a condition is fulfilled, one can control local variables and their gradients to theoretically describe the thermodynamic state of living systems at the micro- and nanoscale. Performing ultralocal experimental manipulations has become possible thanks to the patch-clamp technique, which controls the cell membrane potential, and fluorescence imaging, which monitors molecular concentrations and their intracellular gradients. However, precise temperature gradient control at the micro- and nanoscales has yet to be reliably realized in a living cell.Here, we present a new methodology—microscale control of a temperature gradient profile in aqueous media by a fully optical diamond heater-thermometer in a plug-and-play fiber configuration combined with the patch-clamp technique. In particular, we demonstrate applications of the combined diamond heater-thermometer-patch-clamp approach for fast, reproducible thermal modulation of ionic current from voltage-gated $Na_v1.5$ sodium channels expressed in HEK293 cells and in freshly isolated ventricular mouse cardiomyocytes. Such an approach of manipulating the ultralocal temperature has the potential to uncover previously inaccessible phenomena in various physiological intracellular processes related to the endogenous nanoscale heat sources, such as open ion channels capable of producing Joule heat.

SIGNIFICANCE In this manuscript, we present a new thermometer composed of a diamond (diamond heater-thermometer) designed to be plug-and-play with any patch-clamp system. As explained in this article, this diamond heater-thermometer allows for fast and reproducible thermal modulation at the microscale level of the fast sodium channel $Na_v1.5$ expressed in HEK293 cells and in freshly isolated ventricular mouse cardiomyocytes. Such an approach of manipulating the ultralocal temperature has the potential to uncover previously inaccessible phenomena in various physiological intracellular processes related to the endogenous nanoscale heat sources, such as heat release in the open ion channels, ionic pumps, or during local calcium release processes and muscle contraction.

INTRODUCTION

Studies on the effects of temperature on single cells, tissues, and the whole body show their multifarious roles at all structural levels in living organisms (1–4). The most intriguing are the thermal effects related to intracellular calcium signaling (5–9), thermally induced cell morphology changes (10–12), manipulating embryogenesis via nanoscale temperature control (13), thermal activation of single kinesin molecules (14), thermally induced changes of cell electrical capacitance (15), and thermal activation of thermo-transient receptor potential channels (16). The exceptional importance of temperature as a thermodynamic parameter in life is illustrated by the small range of temperature where life forms can exist, just around 100°C, in comparison with the physical range from 0 K to Plank's temperature of around 10^{32} K (17). A more narrow temperature range of

Submitted June 16, 2025, and accepted for publication November 10, 2025.
*Correspondence: jean-sebastien.rougier@unibe.ch or vadim@nanthermix.com
Editor: Guy Genin.
https://doi.org/10.1016/j.bpj.2025.11.014
© 2025 The Authors. Published by Elsevier Inc. on behalf of Biophysical Society.
This is an open access article under the CC BY license (http://creativecommons.org/licenses/by/4.0/).

37°C–42°C was recently identified as the most energy-efficient for processing neural signals (18). The existence of life in such a narrow temperature range coincides with experimental data demonstrating the possibility of artificially creating steep (up to 20°C/μm) steady-state temperature gradients in volumes of aqueous media at the micro- and nanoscale (19,20). This well corresponds to the extremely low thermal conductivity of water (21) under the assumption of Fourier heat transfer phenomena by conduction (17,22).

Progress in experimental research over the last five decades has enabled the ultralocal control of the membrane potential in single cells using the patch-clamp technique (23) and the measurement of ionic/molecular concentrations, as well as their gradients, through fluorescent imaging with specific fluorescent dyes (24). However, precise ultralocal temperature control at the micro- and nanoscale has yet to be achieved through an unambiguous, undoubtedly accurate, and reliable experimental realization in a living cell, particularly regarding fast electrical and thermodynamic processes in ion channels. Multiple established methods of ultralocal heating based on different physical principles have been developed and successfully used in single-cell experiments, as described in (1). Among them are the methods engaging direct infrared laser heating of water (6,15), ultralocal heating of metal nanoparticles (22,25), and methods engaging excellent photothermal efficacy from the interior of the Si mesostructures due to enhanced intrinsic light absorption in amorphous Si (26), combined with reduced thermal conductivity and heat capacity (27).

Ion channels are pore-forming membrane proteins that enable the movement of ions through an ion-impermeable lipid bilayer and are crucial for numerous biological processes. As with other proteins, temperature has a dramatic influence on the biophysical properties of all ion channels (28–30). The patch-clamp technique enabled the investigation of these biophysical properties (e.g., current amplitude, channel kinetics) (31). Many of those investigations are performed at nonphysiological temperatures (22°C–23°C) for technical purposes (e.g., ensuring the stability of the recording as the fluidity of the lipid bilayer increases with temperature). Although heating devices (e.g., chamber perfusion using the Peltier approach (32–34)) have been developed to investigate ion channels at physiological temperatures, their main limitations are 1) the absence of local and precise increase of temperature leaving the membrane integrity of cells surrounding the investigated one "intact" for further recordings; 2) the inability of increasing the temperature within milliseconds does not enable the investigation of biophysical properties of "fast ion channels," such as the voltage-gated sodium channels family; and 3) the unreliability of the recorded parameters due to the inability of existing methods to apply different temperatures at different times to the same cell (due to the relaxation on a millisecond timescale to achieve a thermodynamic equilibrium).

Here, we present a new and reliable methodology for controlling the temperature gradient profile in aqueous media at the microscale using an all-optical diamond heater-thermometer (DHT), whose basic principles were described previously in (19,20), fully integrated with an optical fiber to allow for a seamless plug-and-play combination with patch-clamp whole-cell electrical recordings. Such an approach enabled us to demonstrate experimentally fast, reproducible, and safe modulation of the biophysical properties of 1) the fast voltage-gated sodium channel expressed either in a heterologous expression system or in native cardiomyocytes and 2) the ventricular cardiac action potential.

MATERIALS AND METHODS

The diamond heater-thermometer device

A fully fiber-coupled version of the DHT device was used. It consisted of a glass pipette with a micron-scale diamond containing embedded temperature-sensing silicon-vacancy (SiV) centers, combining the functions of a microscale heater and thermometer. The device acted in two modes: as a thermometer or as a heater and thermometer simultaneously, depending on the power of laser excitation. The particle contains an amorphous sp2-carbon phase, which is characteristic of the intercrystalline boundaries of diamond polycrystals (35), allowing for increased absorption of laser radiation and enabling it to function as a calibrated thermometer and an ultralocal precision heat source (20). The diamond particle was connected to a tapered optical fiber serving both to guide the excitation light and to collect the fluorescence emitted by the SiV centers. The excitation was provided by a fiber-coupled laser (520 nm, CNI lasers), and a fiber-coupled spectrometer (ATP5200, OptoSky Photonics) was used to detect the fluorescence peak. The fibers were connected in a control unit using a fiber splitter (Thorlabs), ensuring that a single optical fiber end connection to the DHT was necessary. The simplicity of the DHT in the single fiber-coupled configuration ensures its compatibility with most existing laboratory setups, such as the Zeiss Axio Z1 microscope used in this work, without requiring any modifications to the optical paths. The solid-state nature of the DHT guarantees the absence of photobleaching, allowing long experimental protocols of thermal stimulation applied to individual living cells. Being fully optical, the method does not produce electromagnetic noise hindering patch-clamp recordings.

Cell line preparation

Human embryonic kidney (HEK293) cells stably expressing the human voltage-gated sodium channel $Na_v1.5$ were cultured in DMEM (Gibco, Basel, Switzerland) supplemented with 10% FBS, 0.5% penicillin, Zeocin (200 μg/mL), and streptomycin (10,000 U/mL) at 37°C in a 5% CO_2 incubator.

Isolation of mouse ventricular myocytes

All animal experiments were performed according to the Swiss Federal Animal Protection Law and approved by the Cantonal Veterinary Administration of Bern. This investigation conforms to the Guide for the Care and Use of Laboratory Animals, published by the US National Institutes of Health (NIH publication no. 85-23, revised 1996).

Single cardiomyocytes were isolated using a modified procedure based on established enzymatic methods. Briefly, mice were deeply anesthetized using a ketamine/xylazine mix (200/20 mg/kg body weight) via intraperitoneal injection. After losing reflexes, hearts were rapidly excised, cannulated, and mounted on a Langendorff column for retrograde perfusion at 37°C. Hearts were rinsed free of blood with a nominally Ca^{2+}-free solution containing (in mM) 135 NaCl, 4 KCl, 1.2 $MgCl_2$, 1.2 NaH_2PO_4, 10 HEPES,

and 11 glucose (pH 7.4) (NaOH adjusted) and subsequently perfused by a solution supplemented with 50 μM Ca^{2+} and collagenase type II (0.5 mg/mL, Worthington, Allschwil, Switzerland) till achieved digestion. After digestion, the atria were removed, and the ventricles were transferred to a nominally Ca2+-free solution, supplemented with 100 μM Ca^{2+}, and cut into small pieces. Single cardiac myocytes were liberated by gentle trituration of the digested ventricular tissue (using a 1-mL pipette with a wide-bore tip) and filtered through a 100- to 180-μm nylon mesh. Ventricular mouse cardiomyocytes were used after an extracellular calcium increase procedure to avoid calcium overload when extracellular solutions were applied in electrophysiology experiments.

Whole-cell electrophysiology

Sodium currents (I_{Na}) were recorded in the whole-cell configuration using a VE-2 amplifier (Alembic Instrument, USA). Borosilicate glass pipettes were pulled to a series resistance of ~2 MΩ. pClamp software, version 8 (Axon Instruments, Union City, CA, USA), was used for recordings. Data were analyzed using pClamp software, version 8 (Axon Instruments), and OriginPro, version 7.5 (OriginLab, Northampton, MA, USA).

I_{Na} in HEK293 cells was carried out using an internal solution containing (in mM) CsCl 60, Cs-aspartate 70, $CaCl_2$ 1, $MgCl_2$ 1, HEPES 10, EGTA 11, and Na_2ATP 5 (pH was adjusted to 7.2 with CsOH). The cells were bathed in a solution containing (in mM) NaCl 50, N-Methyl-D-glucamine (NMDG)-Cl 80, $CaCl_2$ 2, $MgCl_2$ 1.2, CsCl 5, HEPES 10, and glucose 5 (pH was adjusted to 7.4 with CsOH).

I_{Na} in cardiomyocytes was carried out using an internal solution containing (in mM) CsCl 60, Cs-aspartate 70, $CaCl_2$ 1, $MgCl_2$ 1, HEPES 10, EGTA 11, and Na_2ATP 5 (pH was adjusted to 7.2 with CsOH). Cardiomyocytes were bathed in a solution containing (in mM) NaCl 5, NMDG-Cl 125, $CaCl_2$ 2, $MgCl_2$ 1.2, CsCl 5, HEPES 10, and glucose 5 (pH was adjusted to 7.4 with CsOH). Nifedipine (10 μM) and cobalt chloride ($CoCl_2$) (10 μM) were added to the extracellular solution to inhibit calcium currents.

For cardiac action potential (AP) recordings, cardiomyocytes were bathed in a solution containing (in mM) NaCl 140, KCl 5.4, $CaCl_2$ 1.8, $MgCl_2$ 1.2, HEPES 10, and glucose 5 (pH was adjusted to 7.4 with NaOH). Cardiomyocytes were initially voltage-clamped (holding potential −80 mV) and dialyzed with an internal solution containing (in mM) KCl 120, $CaCl_2$ 1.5, $MgCl_2$ 5.5, Na_2ATP 5, K_2-EGTA 5, and HEPES 10 (pH was adjusted to 7.2 with KOH). APs were elicited at 0.5 Hz with rectangular pulses (5 ms at 125% threshold) in current-clamp mode. Elicited APs were allowed to stabilize before one or more sequences of ~1 min each were acquired from each cell. AP recordings were digitized at a sampling frequency of 20 kHz. Electrophysiological data were analyzed offline, where the resting membrane potential, time to maximum amplitude, and AP durations at 30%, 50%, and 90% repolarization were averaged from each sequence of APs.

The same DHT probe has been used with the HEK293 cell line and cardiomyocytes.

RESULTS

The experiments described here were performed using the setup shown in Fig. 1 *a*, which consisted of an inverted optical microscope, a patch-clamp module, and a DHT device (as described in the materials and methods section). HEK293 cells stably expressing the human voltage-gated sodium channel $Na_v1.5$ were patch-clamped (Fig. 1 *b*), and the sodium current was recorded. During the recording, heat pulses of 30 s were applied to the tip of the fiber-coupled DHT pipette to modulate the biophysical properties of the sodium current (peak current and decay time) as depicted in Fig. 1 *d*. Similar experiments were performed in HEK293 cells not expressing $Na_v1.5$ channels (wild-type HEK293 cells) as a negative control (Fig. 1 *c*).

We then varied the laser power delivered to the DHT pipette to obtain heat pulses reaching various peak temperatures of DHT and consequently producing different steady-state temperature profiles in the cell (Fig. 2). The slope of the temperature profile depends on the laser power delivered to the heat source, its size, and the distance between DHT and the cell (in general around 5–10 micrometers), as discussed in (19,22). With the increased temperature, as anticipated, the sodium peak current values increased, and the current decay times decreased with the increased temperature (Fig. 2). Such effects were observed only in cells stably expressing the voltage-gated sodium channel $Na_v1.5$ and not in the wild-type (see Fig. S1). It is important to note that the laser illumination had little to no effect on those biophysical properties (Fig. S2). Only the laser light absorbed by the diamond particle's surface at the tip of the DHT pipette,

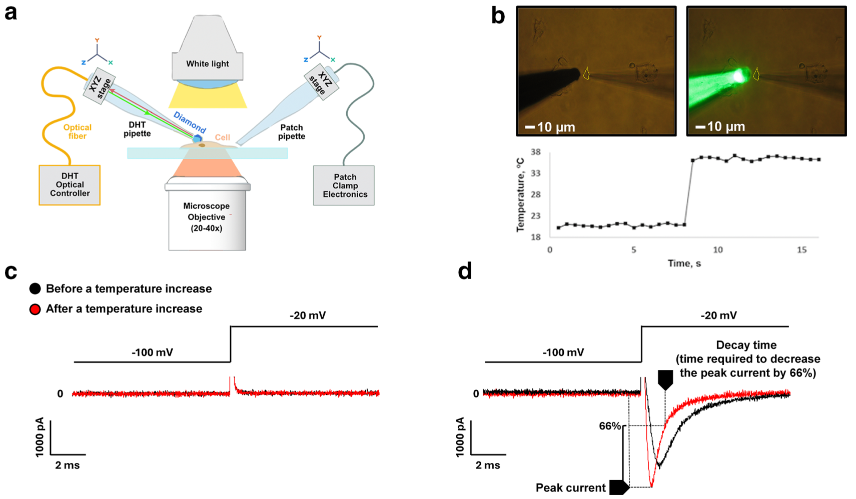

FIGURE 1 Experimental design. (*a*) Schematic representation of a combined DHT patch-clamp setup. (*b*) Microscope image of a clamped HEK293 cell (*yellow dashed line*) with an approaching DHT pipette. (*c*) Whole-cell patch-clamp recording of the ionic current through the cell membrane in the wild-type cells, and (*d*) in cells stably expressing the voltage-gated sodium channel $Na_v1.5$. The artifact of stimulation observed when the membrane voltage is modified has been cut for space purposes.

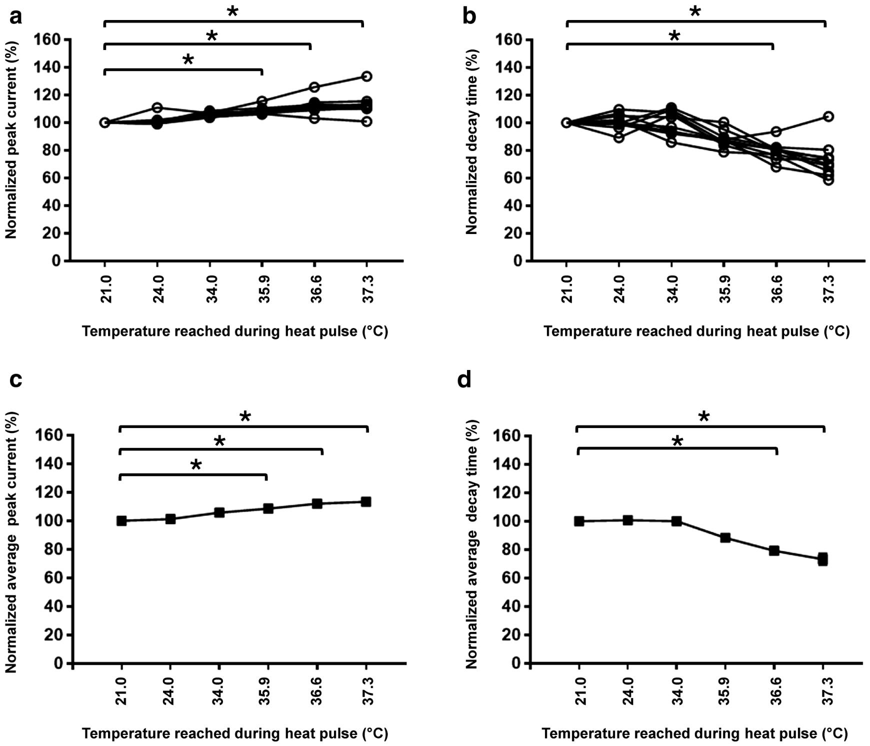

FIGURE 2 Effect of local heat pulse from the DHT. (*a* and *c*) Peak current increased with the increased temperature of the pulse. (*b* and *d*) Decay time decreased when the temperature of the pulse increased. These effects are observed only in cells stably expressing the voltage-gated sodium channel $Na_v1.5$ and not in wild-type cells. $n = 10$ cells. $^*p < 0.05$ versus 21.0°C. (*a* and *b*) Each line, connecting a circle, corresponds to the modification of the parameter for different temperatures applied to a single cell. (*c* and *d*) Average peak (*c*) and decay time (*d*) of the cells represented in (*a*) and (*b*), respectively. The SEMs are not visible because they are too small.

producing local heat, played an observable role. The exact temperature of the heat pulse can be measured in real time, which is one of the key advantages of the DHT system compared with a more standard direct laser heating. Another advantage is the reduced photodamage to living cells, as only several milliwatts of laser power are sufficient to execute thermal stimulation compared with hundreds of milliwatts required by a conventional infrared laser heating approach.

To further test the reliability and repeatability of the DHT device in modulating the ionic current, we performed active thermal cycling between various temperatures (Fig. 3). Within 15 s after modulating the temperature from 19°C to 36°C, we observed that the normalized average peak current increased by ~15% (Fig. 3 *a*). An inverse effect of ~20% change is observed with a similar heat pulse on the average normalized decay time (Fig. 3 *b*). Interestingly, this modulation is reproducible, highlighting the absence of harmful effects of this approach on the cell (Figs. 3 *c* and 3 *d*).

One of the key features of the DHT device is its ability to selectively apply an ultralocal temperature profile owing to its microscopic size. We therefore decided to use different temperatures (36°C and 55°C) in various orders to modulate the peak current. This is hardly feasible with a classical approach due to the time required for thermal dissipation after switching to a new temperature. As shown in Fig. 3, the peak current increase and the decay time decrease observed at 36°C and 55°C are accurately and reproducibly observed independently of the order in which those temperatures were applied. This indicates that the DHT device induced a rapid temperature change due to its ultralocal application and rapid thermal relaxation (on a millisecond timescale) (Figs. 3 *e* and 3 *f*). Thanks to these properties, the heat pulse applied to the cell can be relatively short, and increasing the temperature to 55°C for a short time to record the sodium current does not affect cell viability.

In parallel to experiments with HEK293 cells, we obtained freshly isolated adult ventricular mouse cardiomyocytes and recorded sodium current from their endogenously expressed $Na_v1.5$ channels. We observed similar thermal modulations in the peak sodium current and the decay time in cardiac cells without damaging the surrounding ones (Fig. 4). The increase in peak current amplitude in cardiomyocytes is significantly higher than in HEK293 cells expressing $Na_v1.5$ channels due to the higher sensitivity to temperature of these cardiac cells.

Then, cardiac action potentials were recorded before and during a heat pulse to assess the effect of heat on various parameters (Figs.5 *a* and 5 *b*). As observed in Fig. 5 *a* and represented in Figs. 5 *d* and 5 *g*, a heat pulse induced a significant decrease in the time to maximum amplitude and increase of the APD90 (Fig. 5). In addition, the upstroke velocity was increased, which reflects the increase in sodium current observed in Fig. 4 *a* (Fig. 5 *h*). However, neither the resting membrane potential nor the APD30 and APD50 were significantly different between 21.0°C and 35.9°C (Figs. 5 *c*, 5 *e*, and 5 *f*).

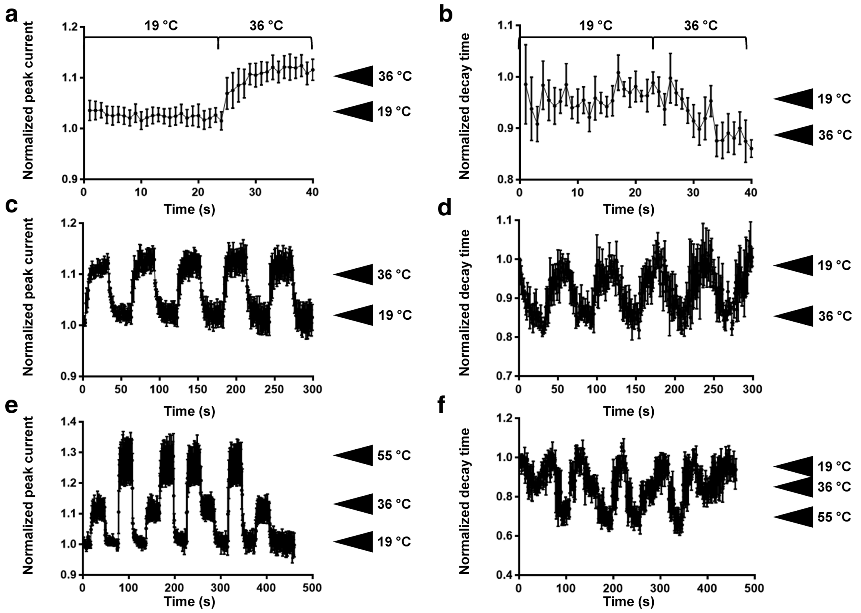

FIGURE 3 Repeatability and reproducibility of the thermal modulation of sodium current. (*a*) Normalized average peak current ($n = 6$) and (*b*) normalized average decay time ($n = 5$) modulation by a heat pulse from 19°C to 36°C. (*c* and *d*) Repeatability of the thermal modulation of the peak current (*c*) and decay time (*d*). Normalized average peak current (*e*) and decay time (*f*) modulation ($n = 3$) by different temperatures (36°C and 55°C), showing the reproducibility of this modulation independently of the sequence of temperature steps applied. The increased noise observed at the end of the decay-time measurements (*d*) is due to seal instability.

DISCUSSION

Comparison of published ion channel data and implications for future experiments

Our group investigated the modulation of the biophysical properties of $Na_v1.5$ wild-type channels by increasing the temperature several years ago using a similar approach (patch-clamp setup and cell line), but with a heating perfusion system rather than the DHT technique (34). No significant difference was observed between our previous data and the data presented in this manuscript regarding the effect on the peak current measured using the whole-cell configuration. However, the decrease in decay time appears smaller in this study than in the one using the heating perfusion system suggesting that temperature was partially heterogeneous (34).

The time required in our previous publication to reach thermal stability and perform the different recordings was approximately 1 min (34), whereas with the DHT approach, this value is closer to 15–25 s. One explanation for this difference may be the position of the heating source. Heating perfusion systems are generally challenging to position close to the cells, as the perfusion of the solution damages the cells (stretching forces). In contrast, the DHT may be more closely positioned to the cell without affecting its viability and, therefore, reduce the time required to heat the cell to the desired temperature. In addition, the time required to reach the requested temperature at the tip of the DHT probe is in the range of milliseconds (36), which is significantly shorter than the time needed to measure and stabilize the current (~seconds). This delay can be explained by the time required to warm the entire water environment surrounding the cell, and it is also observed when using a Peltier device to heat a water bath. Such a delay may make it difficult to investigate the limits of temperature-dependent biophysical modulations that occur at a subsecond timescale. The ease of positioning the DHT close to the target, without affecting the stability of the seal or the noise, as in the case with the heating perfusion system, may help investigate those parameters not only in a whole-cell configuration but also in a single-channel recording experiment using the inside-out and outside-out configurations, which may provide more insight into the bandwidth of thermal modulation in ion channels.

Recording biophysical properties of ion channels at physiological temperatures or higher is a "must-have." A plethora of investigations have highlighted the consequences of hyperthermia in organ dysfunction, such as in the heart (the Brugada syndrome, heat stroke, and heat exhaustion), and the brain (neurological and cognitive alteration), which are probably due to the alteration of ion channel function at high temperatures (6,34). For example, ion channels from the transient receptor potentials family exhibit an eightfold increase in current density with a 10°C increase in temperature (37). Nevertheless, such recordings face multiple technical challenges. The ultralocal temperature controller presented in this manuscript will address many of these challenges and open new avenues for investigating local temperature fluctuations and their implications for ion channel function.

Relevance of modulating ultralocal thermodynamics

Currently, temperature is typically viewed as a macroscale parameter that depends solely on the macroscale heat production and heat conduction within the tissue or body of a

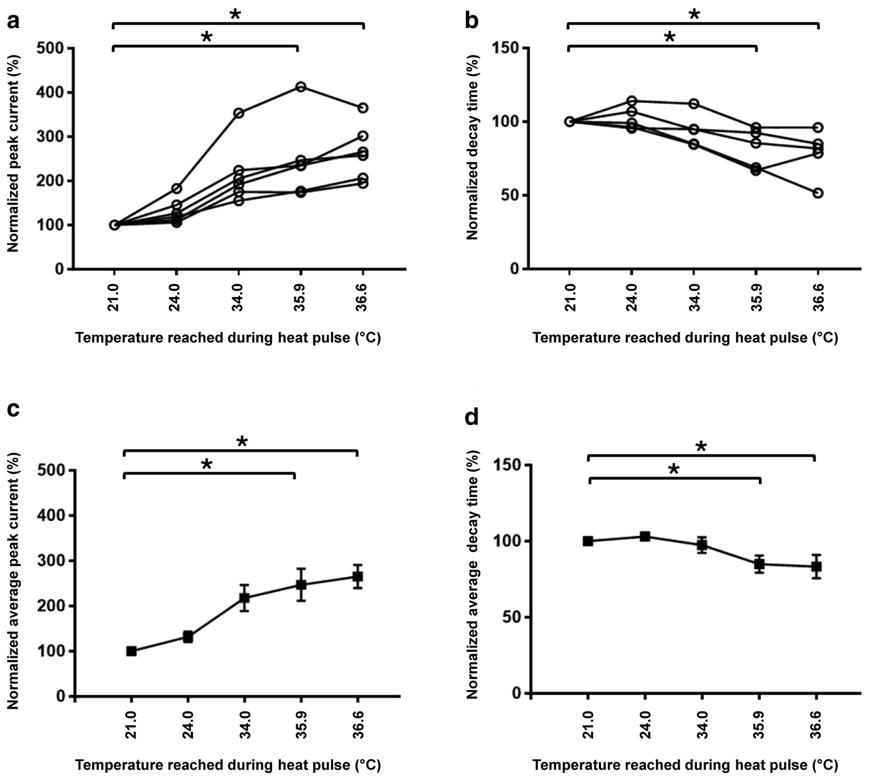

FIGURE 4 Effect of local heat pulse from the DHT on the peak sodium current (*a* and *c*) and decay time (*b* and *d*) extracted from the corresponding patch-clamp recordings using freshly isolated murine adult ventricular cardiomyocytes. $N = 5–6$ cells. $*p < 0.05$ versus 21.0°C. (*a* and *b*) Each line, connecting a circle, corresponds to the modification of the parameter for different temperatures applied to a single cell. (*c* and *d*) Average peak (*c*) and decay time (*d*) of the cells represented in (*a*) and (*b*), respectively. A few SEMs are not visible because they are too small.

multicellular organism, or as an influence of external, usually slowly changing, environmental temperature. Nevertheless, for researchers working in the field of ion channels, the questions related to the ultralocal thermodynamic effects that lie behind the highly probable Joule heat release on open ionic channels are of primary interest, as they are currently dramatically underestimated (38). The heat conductivity coefficient of intracellular media is five to seven times smaller than that of water (39,40); therefore, one can imagine the presence of steep nano- and microscale temperature gradients for both artificial micro- and nanoscale heat sources, as well as for probable endogenous intracellular "hot spots" such as mitochondria, ion pumps, and ion channels. For example, an open ion channel operating under electrical potential on cellular membranes is one of the most intriguing cases of suspected intracellular ultralocal heat sources. Remarkably, up to several tens of degrees temperature rise due to Joule heat release was calculated using nanoscale hydrodynamic modeling of ion velocity distributions accelerating in the ion channels (38). Moreover, since Lars Onsager discovered reciprocal relations between different forces and flows (41,42)), such as the conduction of heat, electrical currents, and diffusion (e.g., Peltier and Seebeck, or Dufour and Soret pair-reciprocal phenomena), the introduction of ultralocal temperature clamp/control method in aqueous media may uncover the previously hidden microscale effects of energy transformation processes in living cells bound to electrochemical potential, for example (42,43).

Therefore, the existing paradigm, which neglects the ultralocal effects of intrinsic cellular thermodynamics, must be updated. The updated view should also consider temperature as an intracellular ultralocal variable at the micro- and nanoscale. The precise control of temperature as an ultralocal variable assigned to the different micro- and nanoscale volumes in aqueous media in such a nonequilibrium thermodynamic system as the living cell should rely on the notion of local equilibria (17,22,44). Simple physical estimations in line with (22) justify that there are no theoretical problems in defining the local temperature for an elementary intracellular volume down to $\sim$100 nm^3. Nevertheless, on the way to this new paradigm, at least two serious problems existed. The first issue is the apparent deficiency of simple, reliable experimental methods for micro- and nanoscale temperature measurements and control in aqueous media near and inside living cells, as discussed in (1,4,19,22). The second concern is the widespread doubt regarding the validity of using correct temperatures in micro- and nanoscale aqueous volumes. However, the presented approach of manipulating ultralocal intracellular temperatures in microscale aqueous volumes has no theoretical or technical limitations to be scaled down to the nanoscale with an appropriate size of the DHT, which may allow for uncovering previously inaccessible effects in various physiological intracellular processes related to endogenous heat sources (e.g., heat release in the open ion channels (38), ionic pumps (45), uncoupled mitochondria (46), and local calcium release processes (6,7), and muscle contraction (47,48)).

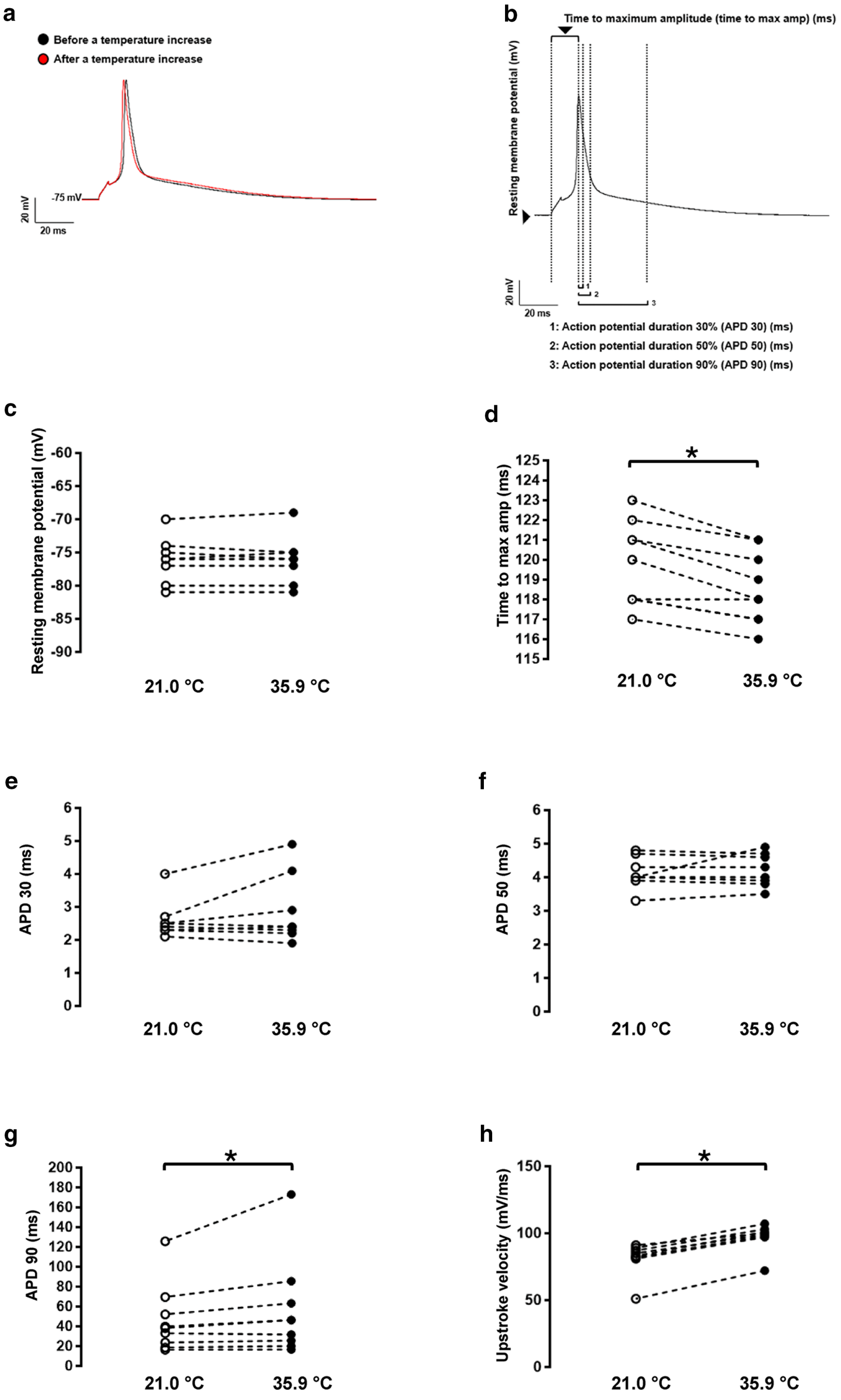

FIGURE 5 Murine adult ventricular cardiac action potential recordings showing the effect of a heat pulse (*a*) and the different biophysical parameters investigated (*b*). Only the time to maximum amplitude (max amp; *d*) and the 90% action potential duration (APD90; g) are significantly different comparing the room temperature (*white circle*, t° = 21.0°C) to heat pulse conditions (*black circles*, t° = 35.9°C; $n = 6$ cells; *$p < 0.05$ versus 21.0°C).

CONCLUSIONS

In conclusion, we present a new methodology for microscale temperature control in aqueous media, enabled by the DHT in a fully optical fiber-based configuration, combined with the patch-clamp technique. Using this approach, we have demonstrated applications for the local, fast, and reproducible thermal modulation of ionic current from voltage-gated $Na_v1.5$ sodium channels present in cardiomyocytes and overexpressed in heterologous expression systems. Moreover, the presented approach of manipulating ultralocal temperatures may open a new avenue of investigations. The potential feasibility of the DHT to modulate temperature not only at the microscale level, as presented in this manuscript, but also at the nanoscale may uncover previously inaccessible effects in various intracellular

physiological processes related to endogenous nanoscale heat sources. Moreover, thanks to the plug-and-play configuration of the demonstrated fiber-coupled DHT, its ease of use, and its versatility, such a technique can become part of a standard toolbox for electrophysiology, calcium imaging, and experimental life sciences in general.

Statistical analysis

Data are represented as means ± SEM. Statistical analyses were performed using Prism7 GraphPad software. Due to the small sample size, a Wilcoxon signed-rank test was used to compare the two groups only (Figs.5 *c*, 5 *d*, 5 *e*, 5 *f*, 5 *g*, and 5 *h*). For multiple comparisons (Figs. 2 and 4), an ANOVA (Friedman) test with a Dunn's multiple comparison test was performed to compare 21.0°C with the other temperatures. $p < 0.05$ was considered significant.

ACKNOWLEDGMENTS

The Institute of Biochemistry and Molecular Medicine, Department of Physiology, University of Bern, thanks NanThermix SA for providing the DHT setup.

We thank Dr. Sarah Vermij for her valuable comments and proofreading on this manuscript.

The Swiss National Science Foundation funded this work (SNF 310030_215274) to H.A. This work was supported by the Human Frontier Science Program RGP0047/2018 (to V.Z.).

AUTHOR CONTRIBUTIONS

J.-S.R., E.G., J.K., V.Z., and H.A.: conceptualization. J.-S.R. and S.G.: investigation. J.-S.R. formal analysis. J.-S.R., E.G., J.K., and V.Z.: writing – original draft.

DECLARATION OF INTERESTS

The authors declare no competing interests.

SUPPORTING MATERIAL

Supporting material can be found online at https://doi.org/10.1016/j.bpj.2025.11.014.

REFERENCES

1. Sistemich, L., and S. Ebbinghaus. 2024. Heat application in live cell imaging. *FEBS Open Bio.* 14:1940–1954.
2. Oyama, K., S. Ishii, and M. Suzuki. 2022. Opto-thermal technologies for microscopic analysis of cellular temperature-sensing systems. *Biophys. Rev.* 14:41–54.
3. Suzuki, M., C. Liu, …, T. Yamazawa. 2023. Trans-scale thermal signaling in biological systems. *J. Biochem.* 174:217–225.
4. Suzuki, M., and T. Plakhotnik. 2020. The challenge of intracellular temperature. *Biophys. Rev.* 12:593–600.
5. Tseeb, V., M. Suzuki, …, S. Ishiwata. 2009. Highly thermosensitive Ca dynamics in a HeLa cell through IP(3) receptors. *HFSP J.* 3:117–123.
6. Oyama, K., V. Zeeb, …, M. Suzuki. 2022. Heat-hypersensitive mutants of ryanodine receptor type 1 revealed by microscopic heating. *Proc. Natl. Acad. Sci. USA.* 119:e2201286119.
7. Morrissette, J. M., J. P. G. Franck, and B. A. Block. 2003. Characterization of ryanodine receptor and Ca2+-ATPase isoforms in the thermogenic heater organ of blue marlin (Makaira nigricans). *J. Exp. Biol.* 206:805–812.
8. Oyama, K., A. Mizuno, …, S. Ishiwata. 2012. Microscopic heat pulses induce contraction of cardiomyocytes without calcium transients. *Biochem. Biophys. Res. Commun.* 417:607–612.
9. Itoh, H., K. Oyama, …, S. Ishiwata. 2014. Microscopic heat pulse-induced calcium dynamics in single WI-38 fibroblasts. *Biophysics.* 10:109–119.
10. Oyama, K., V. Zeeb, …, S. Ishiwata. 2015. Triggering of high-speed neurite outgrowth using an optical microheater. *Sci. Rep.* 5:16611.
11. Black, B., V. Vishwakarma, …, S. Mohanty. 2016. Spatial temperature gradients guide axonal outgrowth. *Sci. Rep.* 6:29876.
12. Oyama, K., T. Arai, …, S. Ishiwata. 2015. Directional bleb formation in spherical cells under temperature gradient. *Biophys. J.* 109:355–364.
13. Choi, J., H. Zhou, …, M. D. Lukin. 2020. Probing and manipulating embryogenesis via nanoscale thermometry and temperature control. *Proc. Natl. Acad. Sci. USA.* 117:14636–14641.
14. Kawaguchi, K., and S. Ishiwata. 2001. Thermal activation of single kinesin molecules with temperature pulse microscopy. *Cell Motil Cytoskeleton.* 49:41–47.
15. Shapiro, M. G., K. Homma, …, F. Bezanilla. 2012. Infrared light excites cells by changing their electrical capacitance. *Nat. Commun.* 3:736.
16. Patapoutian, A., A. M. Peier, …, V. Viswanath. 2003. ThermoTRP channels and beyond: mechanisms of temperature sensation. *Nat. Rev. Neurosci.* 4:529–539.
17. Kondepudi, D., and I. Prigogine. 1998. Modern Thermodynamics, 1st ed. Wiley.
18. Yu, Y., A. P. Hill, and D. A. McCormick. 2012. Warm body temperature facilitates energy efficient cortical action potentials. *PLoS Comput. Biol.* 8:e1002456.
19. Romshin, A. M., V. Zeeb, …, I. I. Vlasov. 2021. A new approach to precise mapping of local temperature fields in submicrometer aqueous volumes. *Sci. Rep.* 11:14228.
20. Romshin, A. M., V. Zeeb, …, I. I. Vlasov. 2023. Nanoscale thermal control of a single living cell enabled by diamond heater-thermometer. *Sci. Rep.* 13:8546.
21. Weast, R. C., M. J. Astle, and W. H. Beyer. 1986. Handbook of Chemistry and Physics, 67 ed. CRC Press.
22. Zeeb, V., M. Suzuki, and S. Ishiwata. 2004. A novel method of thermal activation and temperature measurement in the microscopic region around single living cells. *J. Neurosci. Methods.* 139:69–77.
23. Hamill, O. P., A. Marty, …, F. J. Sigworth. 1981. Improved patch-clamp techniques for high-resolution current recording from cells and cell-free membrane patches. *Pflugers Arch.* 391:85–100.
24. Poenie, R. Y. T. M. 1986. Fluorescence ratio imaging: a new window into intracellular ionic signaling. *Trends Biochem. Sci.* 11:450–455.
25. Carvalho-de-Souza, J. L., J. S. Treger, …, F. Bezanilla. 2015. Photosensitivity of neurons enabled by cell-targeted gold nanoparticles. *Neuron.* 86:207–217.
26. Tanaka, K., E. Maruyama, …, H. Okamoto. 1999. Amorphous Silicon, 1st ed. Wiley.
27. Jiang, Y., J. L. Carvalho-de-Souza, …, B. Tian. 2016. Heterogeneous silicon mesostructures for lipid-supported bioelectric interfaces. *Nat. Mater.* 15:1023–1030.
28. Chowdhury, S., B. W. Jarecki, and B. Chanda. 2014. A molecular framework for temperature-dependent gating of ion channels. *Cell.* 158:1148–1158.
29. Cesare, P., A. Moriondo, …, P. A. McNaughton. 1999. Ion channels gated by heat. *ProcNatlAcadSciUSA.* 96:7658–7663.

30. Bassetto, C. A. Z., Jr., B. I. Pinto, …, F. Bezanilla. 2023. Ion channel thermodynamics studied with temperature jumps measured at the cell membrane. *Biophys. J.* 122:661–671.

31. Hille, B. 1978. Ionic channels in excitable membranes. Current problems and biophysical approaches. *Biophys. J.* 22:283–294.

32. Dorst, M., and K. Vervaeke. 2024. A low-cost perfusion heating system for slice electrophysiology. *Sci. Rep.* 14:28521.

33. Harberts, J., M. Kusch, …, R. H. Blick. 2020. A Temperature-Controlled Patch Clamp Platform Demonstrated on Jurkat T Lymphocytes and Human Induced Pluripotent Stem Cell-Derived Neurons. *Bioengineering (Basel).* 7:46.

34. Keller, D. I., J. S. Rougier, …, H. Abriel. 2005. Brugada syndrome and fever: genetic and molecular characterization of patients carrying SCN5A mutations. *Cardiovasc. Res.* 67:510–519.

35. Romshin DGP, A. M., A. S. Altakhov, …, V. P. Filonenko. 2023. Temperature characteristics of “silicon-vacancy” luminescent centers in diamond particles synthesized by various methods. *Opt Spectrosc.* 131:141.

36. Romshin, A. M., N. A. Aseyev, …, P. M. Balaban. 2024. Rapid neurostimulation at the micron scale with an optically controlled thermal-capture technique. *Biomater. Sci.* 13:250–260.

37. Kashio, M. 2025. Thermo-TRP regulation by endogenous factors and its physiological function at core body temperature. *Physiol. Rep.* 13:e70164.

38. Chen, D. P., R. S. Eisenberg, …, C. W. Shu. 1995. Hydrodynamic model of temperature change in open ionic channels. *Biophys. J.* 69:2304–2322.

39. Sotoma, S., C. Zhong, …, M. Suzuki. 2021. In situ measurements of intracellular thermal conductivity using heater-thermometer hybrid diamond nanosensors. *Sci. Adv.* 7:eabd7888.

40. Lu, K., T. Wazawa, …, T. Nagai. 2022. Intracellular Heat Transfer and Thermal Property Revealed by Kilohertz Temperature Imaging with a Genetically Encoded Nanothermometer. *Nano Lett.* 22:5698–5707.

41. Onsager, L. 1931. Reciprocal Relation in Irreversible Processes. I. *Phys. Rev.* 37:405–426.

42. Miller, D. G. 1960. Thermodynamics of Irreversible Processes. The Experimental Verification of the Onsager Reciprocal Relations. *Chem. Rev.* 60:15–37.

43. Guggenheim, E. A. 2002. Modern Thermodynamics by the Methods of Willard Gibbs. *J. Phys. Chem.* 38:713.

44. Landau, L. D., and E. M. Lifshitz. 1964. Statistical Physics. Course of Theoretical Physics. Nauka Press.

45. de Meis, L. 2003. Brown adipose tissue Ca2+-ATPase: uncoupled ATP hydrolysis and thermogenic activity. *J. Biol. Chem.* 278:41856–41861.

46. Romshin, A. M., A. A. Osypov, …, I. I. Vlasov. 2022. Heat Release by Isolated Mouse Brain Mitochondria Detected with Diamond Thermometer. *Nanomaterials.* 13:98.

47. Reggiani, C., E. J. Potma, …, G. J. Stienen. 1997. Chemo-mechanical energy transduction in relation to myosin isoform composition in skeletal muscle fibres of the rat. *J. Physiol.* 502:449–460.

48. Periasamy, M., J. L. Herrera, and F. C. G. Reis. 2017. Skeletal Muscle Thermogenesis and Its Role in Whole Body Energy Metabolism. *Diabetes Metab. J.* 41:327–336.

Biophysical Journal, Volume *125*

Supplemental information

All-optical diamond heater-thermometer enables versatile and reliable thermal modulation of ion channels at the single-cell level

Jean-Sébastien Rougier, Eugene Glushkov, Sabrina Guichard, Jan Kucera, Vadim Zeeb, and Hugues Abriel

All-optical Diamond Heater-Thermometer enables versatile and reliable thermal modulation of ion channels at the single-cell level

Jean-Sébastien Rougier[1*], Eugene Glushkov[2,3], Sabrina Guichard[1], Jan Kucera[4], Vadim Zeeb[2*], Hugues Abriel[1]

[1] Institute of Biochemistry and Molecular Medicine, University of Bern, Bern, Switzerland

[2] NanThermix SA, EPFL Innovation Park, 1015 Lausanne, Switzerland

[3] Laboratory of Nanoscale Biology (LBEN), EPFL, 1015 Lausanne, Switzerland

[4] Department of Physiology, University of Bern, Bern, Switzerland

*Corresponding email: jean-sebastien.rougier@unibe.ch, vadim@nanthermix.com

Supplementary

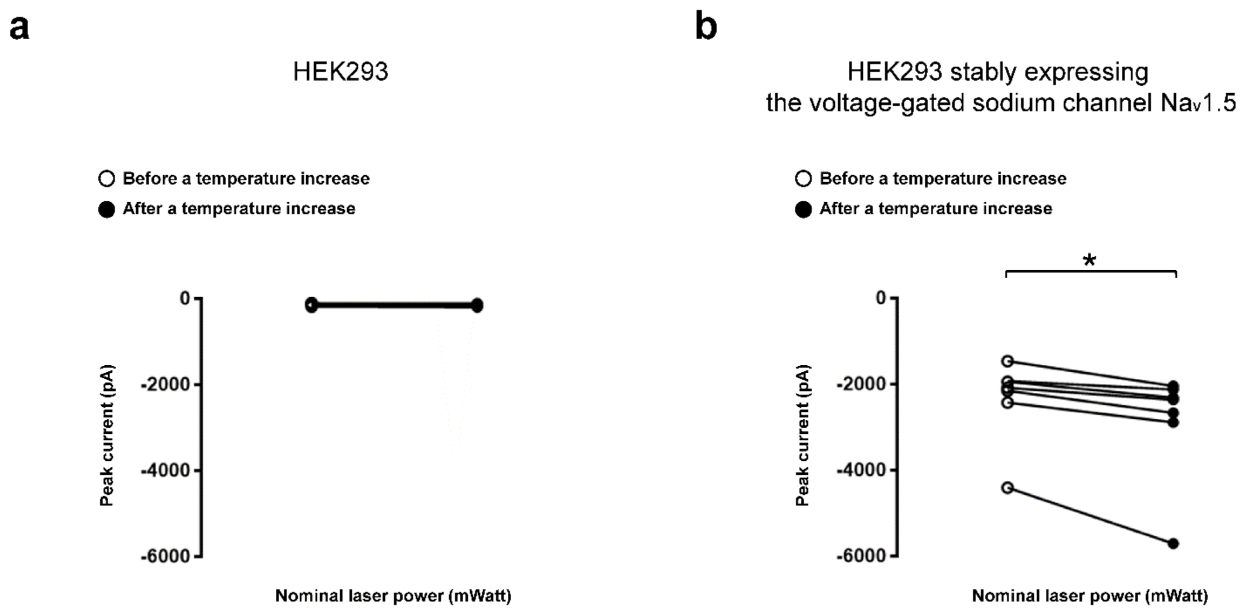

Figure S1. Only the voltage-gated sodium channels Nav1.5 are modulated by the local heat pulse: a) wild-type cells; b) cells stably expressing the voltage-gated sodium channels Nav1.5.

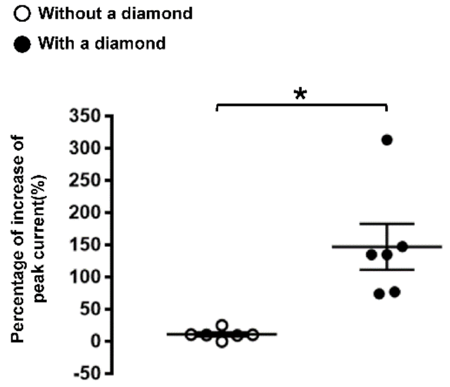

Figure S2. Laser illumination does not affect the biophysical properties of the Nav1.5 channel, as shown by the difference in the peak current increase when a pipette without (white circle) and with (black circle) a diamond is used at a laser power of 40 mW (*; $p < 0.05$).
